# Mental Health of Health and Medical Students during the COVID-19 Pandemic: National Studies

**DOI:** 10.1192/j.eurpsy.2023.69

**Published:** 2023-07-19

**Authors:** A. Frajerman

**Affiliations:** ^1^MOODS team, U1178, INSERM; ^2^Service Hospitalo-Universitaire de Psychiatrie de Bicêtre, Université Paris Saclay, Kremlin-Bicêtre; ^3^Institut de psychiatrie et neurosciences de Paris, U1266, INSERM, Paris, France

## Abstract

**Abstract:**

Since the COVID-19 pandemic’s beginning, psychiatrists and searchers have been worried about mental health degradation, especially for caregivers and students. Health students are still students and yet caregivers.

Two national studies were done in 2021. First on all health students from April 4^th^ to May 11^th^ 2021 (during the 3^rd^ lockdown in France, 1 year after the first one). Second only on medical students from May 27^th^ and June 27^th^ 2021. Both used online surveys

In the first, 16,937 health students answered, including 54% of nurse students. Regarding Kessler- 6 scale for psychological distress, 14% had moderate (8–12), and 83% had high (≥13) levels of psychological distress. In multivariate analysis, being unable to isolate themselves and having financial difficulties were associated with an increased risk of

psychological distress. On the opposite, being a man and not living alone were associated with a reduced risk of psychological distress.

In the second, 11,754 participants (response rate: 15.3%) were included. Prevalence of 7-day anxiety symptoms, 7-

day depressive symptoms assessed by Hospitalization Anxiety and Depression Scale (HADS), 12-month MDE (using Composite International Diagnostic Interview- Short Form), and 12-month suicidal thoughts were 52%, 18%, 25%, and 19%, respectively. Burnout syndrome (evaluated by Maslach Burnout Inventory) concerned 64% of clinical students and residents and 30% of preclinical students.

These 2 studies highlighted the elevated level of mental distress in health students, especially medical students in France. Preventive and curative actions are needed to help them.

**Disclosure of Interest:**

None Declared

